# Proteomics of rice seed germination

**DOI:** 10.3389/fpls.2013.00246

**Published:** 2013-07-09

**Authors:** Dongli He, Pingfang Yang

**Affiliations:** Pingfang Yang, Key Laboratory of Plant Germplasm Enhancement and Specialty Agriculture, Wuhan Botanical Garden, Chinese Academy of SciencesWuhan, China

**Keywords:** proteomics, seed germination, rice, metabolism, post-translational modification

## Abstract

Seed is a condensed form of plant. Under suitable environmental conditions, it can resume the metabolic activity from physiological quiescent status, and mobilize the reserves, biosynthesize new proteins, regenerate organelles, and cell membrane, eventually protrude the radicle and enter into seedling establishment. So far, how these activities are regulated in a coordinated and sequential manner is largely unknown. With the availability of more and more genome sequence information and the development of mass spectrometry (MS) technology, proteomics has been widely applied in analyzing the mechanisms of different biological processes, and proved to be very powerful. Regulation of rice seed germination is critical for rice cultivation. In recent years, a lot of proteomic studies have been conducted in exploring the gene expression regulation, reserves mobilization and metabolisms reactivation, which brings us new insights on the mechanisms of metabolism regulation during this process. Nevertheless, it also invokes a lot of questions. In this mini-review, we summarized the progress in the proteomic studies of rice seed germination. The current challenges and future perspectives were also discussed, which might be helpful for the following studies.

## Introduction

For many plants, seed is essential to reproduce and disperse their progenies. It is also an adaptive strategy for plant survival under stresses. Seed germination, which is an early and crucial stage in plant life cycle, refers to the physiological process starting from the uptake of water by the dry seed and ending with the radicle protrusion (Bewley, 1997). The special water-uptake curve depends on the species and genotype. Seed germination is a complex physiological and biochemical process that involves a series of signal transduction and gene expression regulation. As a major branch of the modern seed biology, studies on seed germination have obtained great progresses in the last two decades. In spite of this, how those complex activities are regulated in a coordinated and sequential manner during germination is largely unknown.

Rice is a monocotyledonous model plant and an important food crop. Rice seed germination could determine its seedling growth and yield to some extent. Plants have evolved fine mechanisms (protect and repair from damage) to ensure the seeds to preserve germination capability. Among them, seasonal dormancy is a common selection for environment tolerance (Footitt et al., 2011). However, most cultivated rice seeds have no or shallow degree of dormancy. In some cultivars, the mature seed could germinate in panicles (named as pre-harvest sprouting or vivipary) encountering suitable climate conditions, which reduces seed yield and quality. Genes related to ABA synthesis has been proved to be involved in the pre-harvest sprouting of rice seed (Fang and Chu, 2008). Interestingly, germination is reversible in some species. The late maturation process of seeds can be re-induced partially at the early stages of germination (Lopez-Molina et al., 2002), which might provide an idea for vivipary preventing. Seed storage is often accompanied with a progressive seed aging and loss of germination vigor even under the “best” storage conditions. Rice seed longevity has been proved to be controlled by several genetic factors (Miura et al., 2002). These problems are the main objectives for the studies on seed germination.

Rice is the first crop with the genome being sequenced (Goff et al., 2002; Yu et al., 2002). The whole genome functional annotation and micro-array analyses development make it an ideal monocotyledonous model system in modern plant biological studies. Depending on the expansion of sequence data, several large-scale-omics including transcriptomic, proteomic, and metabolomic methods were established to investigate the mechanisms of seed germination (Holdsworth et al., 2008). Usually, the abundant storage mRNA in the dry seeds can satisfy the protein *de novo* and instant biosynthesis upon imbibition. Although *de novo* mRNA synthesis can help to ensure the rapidity and uniformity of germination, inhibition of transcription by α-amanitin or actinomycin D could not prevent germination; whereas, blocking the translation with cycloheximide resulted in total inhibition of the germination (Rajjou et al., 2004; He et al., 2011a; Sano et al., 2012). Since the entire complex reactions of seed germination are mainly enforced by different proteins, protein profile analysis might be more precise to clarify this physiological process.

**Figure 1:**
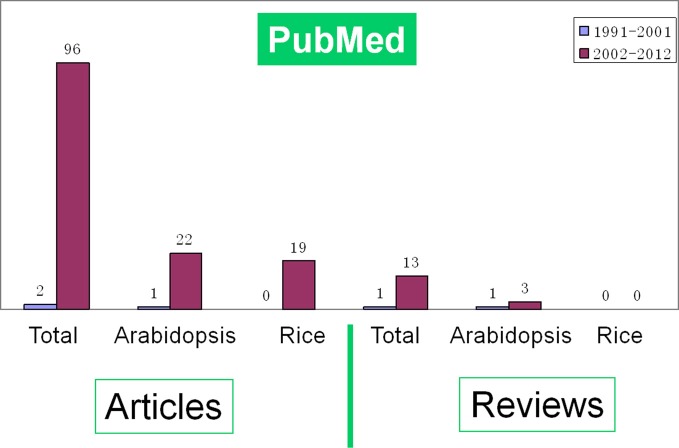
**A survey of the literatures indicates the growing number of proteomic research articles and reviews on seed germination in all species (total), *Arabidopsis* and rice respectively during proteome construction period (1991–2001) and sharply rising period (2002-).** The data was downloaded from PubMed database.

With huge available data of genome information and the development of mass spectrometry (MS) technology, proteomics is exerting great influence in analyzing the dynamic and diverse biological processes. A series of comprehensive reviews have summarized the progresses of proteomics and its impacts on rice (Komatsu et al., 2003; Rakwal and Agrawal, 2003; Agrawal and Rakwal, 2006; Agrawal et al., 2006, 2009; Komatsu and Yano, 2006; Agrawal et al., 2011). A literature survey indicates that the number of proteomic studies on rice seed germination was gradually increased during the early period (1991–2001) and sharply risen in the last decade (2002-; Figure 1). However, there is no review on the proteomics of rice seed germination until now. Comparing with another model plant *Arabidopsis*, rice seems to have much more to be elucidated. Here, we review the progresses in the proteomic study of rice seed germination. Meanwhile, the challenges and future perspectives in this field are also discussed.

## Physiological features of rice seed in germination

**Figure 2:**
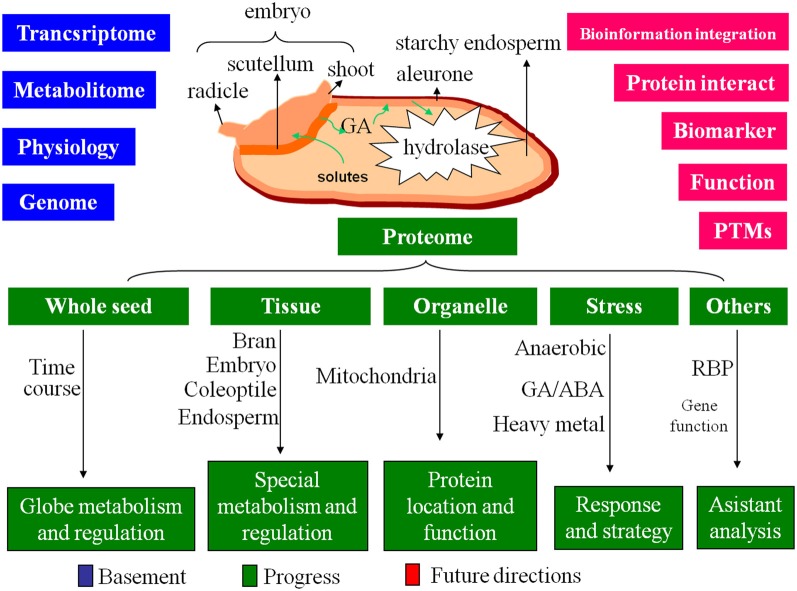
**The basement, progress and future directions of the proteome on rice seed germination.** Based on the great achievements of the genome, transcriptome, metabolitome and physiology, the proteome of rice seed germination obtained progress on multi-level, future directions will focus on the intensive studies of protein interaction, protein biomarker screening, PTMs, individual protein function analysis, and bioinformation integration.

Rice seed has a dominant endosperm for nutrient-storage. The starchy endosperm is surrounded by aleurone layer and neighbored with embryo. Between endosperm and embryo, there is scutellum, a metamorphosis of cotyledon. Embryo and endosperm play different roles in rice seed germination. The embryo contains most of the genetic information that control the germination. Upon imbibition, the substrate and energy starvation will activate the embryo to produce phytohormone [mainly gibberellic acid (GA)]. The GAs can diffuse to aleurone and initiate a signaling cascade that leads to synthesis of α-amylases and other hydrolytic enzymes. These enzymes will then secrete into the endosperm to drive the degradation of storage compounds including starch, lipid and protein for seedling establishment (Jacobsen et al., 1995; Bethke et al., 1997; Figure 2).

**Figure 3:**
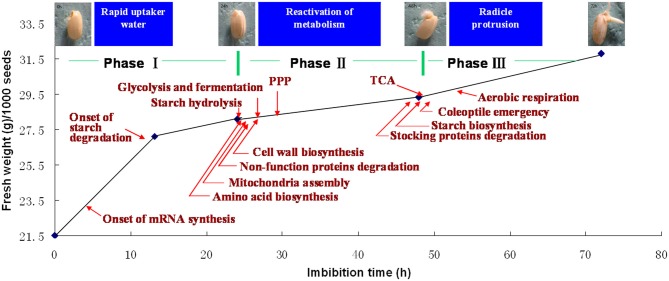
**Sequentially happened activities during rice seed germination.** Upon imbibition, rice seed germination process could be divided into three phases (Yang et al., 2007). Phase I is the first rapid water-uptake period with the onset of mRNA biosynthesis (Howell et al., 2009), phase II is the most important stage for metabolism reactivation, mobilization of reserves, cell structure repair, cell wall loosening, and coleoptile elongation, phase III is another rapid water-uptake stage with TCA and aerobic respiration recovering, cell division initiation, radical protrusion, and initiation of seedling establishment. The picture displayed the rice seed after 0, 24, 48, and 72 h imbibition (provided by Mr. Chao Han).

During seed germination, the increasing of total water content or fresh weight follows a classic triphasic model (Bewley, 1997). When germinating in the distilled water, the rice seed weights increased rapidly during the first 20 h imbibition (phase I), and there is no significant morphology changes. The phase I is followed by a stable plateau stage until 50 h (phase II) during which the coleoptiles elongation could be observed at this stage. Phase III is another rapid water uptake stage accompanying with the protrusion of the radical (Yang et al., 2007; Figure 3). Phase II was usually regarded as the most important stage, because all of the germination required metabolic reactions are reactivated during this period. However, transcriptome of germinating rice seed indicated that the switch may happen even earlier, since a greater proportion of immediate transcript has been identified at this point (Howell et al., 2009).

A series of exogenous and endogenous factors participate in germination regulation, including water, temperature, light, circadian rhythm, and phytohormones (Penfield et al., 2005; Holdsworth et al., 2008). Water is one of the decisive factors, and germination could be arrested under conditions of water deficit (Hundertmark et al., 2011). Rice is one of the few plants that can germinate anaerobically through a rapid elongation of coleoptile (Menegus et al., 1991; Perata et al., 1997). But the radicle could not protrude well under this condition. Once it is switched to the aerobic condition, the radicle can continue to elongate, which suggested that oxygen availability is another determinant factor for true germination (Howell et al., 2007). For Arabidopsis, pre-cooling the moist seed at low temperature (4°C) would stimulate the germination for water-uptake and dormancy-breaking. However, high temperature sprouting is often used to strengthen rice seed germination. To the contrary, low temperature treatment could injure its radicle.

## Progress in proteomics of rice seed germination

Agrawal et al. (2006) have forecasted that rice proteomics is stepping into the second phase that mainly focuses on function analysis and network construction from the first stage characterized by description of proteome profiles. Rice proteomics have also contributed to unveiling the mechanism of the rice seed germination (Table S1; Figure 3).

To give an overall view of the protein mobilization during the rice seed germination, comparative two-dimensional electrophoresis (2-DE) of whole germinating seeds with a time-course sampling were carried out on different rice species (*Oryza sativa* cv. Indica. 9311 and *Oryza sativa* cv. Nipponbare) by Yang and his coworkers (Yang et al., 2007; He et al., 2011b), 148 and 39 proteins were displayed differentially in the process, respectively. The drastic changed patterns of these proteins indicate that germination not only consumes reserves, but also bestows the plant with defenses and rebuilds the morphology. Storage proteins and some seed development- and desiccation-associated proteins were down-regulated; conversely, catabolism-associated proteins were up-regulated with the extension of imbibition time. It deserves to be mentioned that the degradation of seed maturation and desiccation associated proteins (24 h imbibition) mainly occurred a little early than that of storage proteins (48 h imbibition, Figure 3). Since phase II was the most important stage in seed germination, acquiring a comprehensive knowledge of this stage is necessary. Protein profile was obtained from the germinating rice seeds at 24 h after imbibition through 1-DE via liquid chromatography and tandem MS (LC-MS/MS) proteomic shotgun strategy (He et al., 2011a). Totally, 673 proteins sorted into 14 functional groups were identified. Based on the proteomics data, a comprehensive metabolic and regulatory pathway was constructed, which contributed to understanding the metabolic style, regulation of redox homeostasis, and gene expression during rice seed germination. The results also suggested that mobilization of reserves occured during rice seed germination not only by means of central carbon metabolism pathways (glycolysis and TCA cycle) but also by fermentation pathway (Figure 3).

The embryo plays a decisive role during seed germination, so protein profile analysis in the germinating seed embryo may be helpful in unraveling the complex mechanism of this process. Proteins were extracted from 1 or 2 d imbibied embryo of rice seed and analyzed by 2-DE (18 cm IEF tube gel). Sixty differentially expressed proteins were identified and sorted into 10 categories by their functions: metabolism, oxygen-detoxifying, protein processing/degradation, stress/defense, energy and others. This suggested that there are multiple regulations in embryo during seed germination (Kim et al., 2009).

Coleoptile is a protective sheath covering the emerging shoot. For germinating rice seed, the anoxia stress can induce the expression of α-amylase (*RAmy3D*) to degrade the starch and provide substrates for elongating rice seed coleoptiles (Perata et al., 1992). Coleoptile elongation was suppressed in a *cipk15* mutant under submergence and was rescued by adding exogenous sucrose (Lee et al., 2009). Using DIGE and iTRAQ analysis, 140 and 142 differentially expressed proteins were identified from the coleoptiles treated by 4 d of aeration vs. 6 d of anoxia as well as 4 d of aeration with an additional 1 d under anoxia. Proteins related to translation and antioxidant defense were significantly up-regulated in anoxic coleoptiles. Furthermore, accumulation of amino acids (serine, glycine, and alanine), which might be synthesized from glyceraldehyde-3-phosphate or pyruvate, was also detected. This result indicates that amino acid biosynthesis might contribute to anoxia tolerance in cells besides sugar and energy (Shingaki-Wells et al., 2011).

Aleurone is a living tissue surrounding the starchy endosperm and synthesizing key enzymes for germination. The single layer aleurone (unlike multiple-layers in barley) is difficult to be separated in rice seed manually, but the aleurone-rich bran might be selected as a substitute. A complete protein snapshot combined with both gel-based (1-DE and 2-DE) and gel-free (LC-MS/MS) strategies were applied on full-fat or defatted rice bran, 43 unique proteins were identified including signaling/regulation proteins (30%), proteins with enzymatic activity (30%), storage proteins (30%), transfer (5%) and structural (5%) proteins (Ferrari et al., 2009). Yano et al. (2001) had developed a disulfide proteome technique to visualize redox changes in proteins. Rice bran was analyzed by the disulfide proteome technique, and embryo-specific protein 2 (ESP2), dienelactone hydrolase, putative globulin, and globulin-1S-like protein were identified as putative target of thioredoxin, which support the hypothesis that thioredoxin activates cysteine protease with a concurrent unfolding of its substrate during germination (Yano and Masaharu, 2006).

GA and ABA (abscisic acid) are two main phytohormones that antagonistically regulate seed germination by modulating GA- and ABA-responsive functional proteins. GA can promote seed germination and break dormancy, while ABA can induce dormancy at the later phase of seed maturation. GA and ABA pathways might interact to each other by directly inhibiting the synthesis of some proteins or regulating the common target proteins such as DELLA. A proteomic analysis of rice germination under GA and ABA modulation was carried out on embryonic tissue, and 16 notably modulated proteins were identified, including multi-functions: metabolism, oxygen-detoxification, stress/defense functions, protein processing/degradation, signaling and cell wall structure. Western blot and immunolocalization analysis of two differentially expressed protein, rice isoflavone reductase (*OsIFR*) and rice PR10 (*OsPR10*), revealed that both proteins were specifically expressed in the embryo and dramatically down regulated by ABA, which suggested that proteins in the embryo rather than endosperm might be more sensitive to phytohormones (Kim et al., 2008a).

Seed germination provides a good system for studying mitochondria biosynthesis. As an endosymbiotically derived organelle, the typical cristae-rich mitochondria cannot be *de novo* synthesized, but are developed from unstructured double membrane bounded pro-mitochondria. Transcriptome revealed that a greater proportion of transcripts encoding proteins located in the mitochondria peaked early at 1 or 3 h after imbibition (Howell et al., 2009). Coincident with this was the rapid changes in mitochondrial protein content and function. Comparison between embryo mitochondrial protein profiles of dry seed and 48 h imbibed seed, 56 differentially expressed proteins were identified including the outer membrane channel TOM40 and the inner membrane TIM17/22/23 families. Usually, proteins involving import apparatus are very few in the mature mitochondria; the accumulation of these import proteins in the dry seed could operate functions after 2 h imbibition, and then sequentially assemble the components for TCA cycle and electron transport chain (Howell et al., 2006). Anoxic germination also provides a system for oxygen signals studies. Comparison between mitochondrial protein profiles of rice seed germinated for 48 h at anaerobic and aerobic conditions showed that 13 proteins appeared differently in abundance. Although three proteins from the TIM17/22/23 family were 6–14 folds up-regulated under anaerobic conditions, the capacity of the general import pathway was found to be significantly lower in mitochondria under anaerobic conditions than that under aerobic conditions. In this system, import capacity was ultimately dependent on the presence of oxygen; which provided a link between the respiratory chain and protein import apparatus (Howell et al., 2007).

Dry rice seeds contain more than 17,000 stored mRNAs (Howell et al., 2009). The RNA-binding proteins (RBP) perform important role in keeping the stability and regulating the functions of those long-lived mRNAs. Masaki et al. (2008) fractionated RBP in dry rice seeds by single stranded DNA (ssDNA) affinity column chromatography, and revealed three types of RBP in mature seeds: Khomology (KH) domain containing protein, putative RNA- binding protein and glycine-rich RNA-binding protein. The putative RNA-binding protein and glycine rich RNA-binding protein were down-regulated after seeds imbibition, which might promote germination. It is still unknown how the biosynthesis of different proteins happens sequentially during germination. Comparative proteomic analysis on rice seed embryos dissected at 0, 2 and 4 d treated with transcriptional inhibitor actinomycin D (Act D) revealed 20 up-regulated proteins including some carbohydrate metabolism and cytoskeleton formation related proteins. Among them, the translation timing of 8 proteins was clearly later than that of the other 12 proteins. This indicated that the long-lived mRNAs present in dry rice seed are selectively translated depending on the germination phase (Sano et al., 2012). This selective translation machinery also has been observed in maize seeds germination (Jime'nez and Aguilar, 1984).

Because of their sessile character, plants have to confront with many different adverse environments during their whole life. In nature, plant seeds have to overcome different stresses to ensure a survival. These stresses include salt, dehydration, osmotic, and extreme temperature. Currently, with the rapid industrial development, heavy metals, such as copper, cadmium, and arsenic, are also a serious problem in agricultural production. The molecular mechanisms of resistance to these stresses have been systematically conducted in Arabidopsis seed germination (Daszkowska-Golec, 2011). Such studies were also performed in rice during its seed germination. Under copper stress, the germination rate, shoot elongation, plant biomass, and water content all decreased in rice, whereas TBARS (thiobarbituric acid reactive substance) content accumulated quickly. Proteomic analysis revealed that 18 proteins were up-regulated in germinating rice seed under copper stress, including some antioxidant and stress-related proteins such as glyoxalase I, peroxiredoxin, aldose reductase, which suggested that excess coppers could generate oxidative stress. Key metabolic enzymes such as α-amylase and enolase were down-regulated, which indicated excess copper could affect water uptake and reserve mobilization (Ahsan et al., 2007a). Arsenic stress was also reported to decrease α-amylase activity in germinating wheat seeds (Liu et al., 2005). Cadmium also induced the expression of proteins related to defense, detoxification and antioxidant during rice seed germination (Ahsan et al., 2007b). Some low molecular-weight proteins participate in detoxification of heavy metals. Sixteen proteins in the 6–25-kDa range were identified by embryo proteome of rice seeds germinated in presence or absence of 200 mM Cu for 6 days. This study provided the first proteomic evidence that metallothionein and CYP90D2 were Cu-responsive proteins in plants (Zhang et al., 2009).

Proteome analysis has superiority in obtaining specific evidence on the function of designated gene. Komatsu et al. (2005) had investigated Gα (a subunits of heterotrimeric G proteins) protein-regulated proteins in the rice *dwarf1* (d1) mutant. The mutant showed abnormal morphology with shortened internodes, dark green leaves, and small-round grains. While a constitutively active mutant (QL/d1) of the Gα protein produced large round seeds. Using 2-DE, 7 seed embryo proteins were identified down-regulated in the d1 mutant. A receptor for activated C-kinase (RACK) was discovered decreased in d1 mutant but increased in QL/d1. The RACK would disappear after 24 h imbibition, while ABA could promote its expression, which suggested ABA could promotes the expression of both Gα protein and RACK that controls rice seed germination. These data showed that RACK was also regulated by Gα-protein and played an important role in rice germination.

Biomarker hunting is an important purpose for proteomic study; and proteomics is proved to be a powerful tool in screening the biomarker. Probenazole-inducible gene (PBZ1) is a protein marker that was successfully identified by proteomic analysis of rice lesion mimic mutant (spl1) for cell death (Kim et al., 2008b). Phytic acid can form stable complexes with divalent cations and decrease its bioavailability. Comparative proteome analysis of dry seed was performed between the low phytic acid (lpa) rice line (Os-lpa-XS110-1) and its parental line (Xiushui 110, XS-110). Two major differentially expressed proteins, triose phosphate isomerase (TPI) and fructose biphosphatealdolase (FBA) were found to be involved in phytic acid metabolism. These two proteins might be developed as biomarkers for rice seed quality evaluation (Emami et al., 2010). Based on our study (Yang et al., 2007), a starch degradation enzyme α-amylase was sharply increased during rice seed germination. This protein might be an ideal candidate biomarker for rice seed germination.

Seed hereditary is the main factor influencing germination. Hybrid rice cultivars usually have high germination vigor. Comparison of the proteome profiles of mature rice embryo were conducted in hybrid cultivar and its parental lines. Most of the storage proteins exhibited over-dominance, and stress-induced proteins displayed additive effects, which might contribute to abiotic or biotic stress resistance (Ge et al., 2008). In addition to starch, rice endosperm also reserves lots of proteins. Comparative proteomic analysis of endosperm proteins was carried out on two hybrid *indica* cultivars [Liangyoupeijiu (LYP9) and Shanyou63]. Although obviously differences existed in morphology, physiology, and grain quality, the 2-DE profiles displayed that they had nearly the same protein compositions, except for some isoforms of peroxiredoxin, aldose reductase and granule-bound starch synthase were specific in LYP9 or Shanyou63 (Yang et al., 2006).

## Proteins involved in rice seed germination

Upon imbibition, vivid rice seeds should reboot the system activity and mobilize the reserves for germination. Since protein is the real executor of life activities, a series of proteins will participate in the germination process. Some indispensable proteins for germination will be discussed here, such as those related to metabolic, storage, protein synthesis, ROS scavenger, signaling to name a lot.

Without exception, metabolism-related proteins, especially those involved in the carbohydrate metabolic pathways including major and minor carbohydrates metabolism, glycolysis, TCA cycle, fermentation, gluconeogenesis and glyoxylate cycle and pentose phosphate pathway (PPP), were the major group of proteins existing in germinating rice seed (Figure 3). Since starch is the major reserve of the rice endosperm, all the enzymes involved in starch degradation to hexose phosphate were identified in rice seeds at 24 h of germination. Except for α- amylase that was greatly increased in abundance at the late stage of phase II, all the other enzymes keep constant in the whole germination process (Yang et al., 2007; He et al., 2011a), which suggested that the starch degradation metabolism is vigorous at the phase II. The hexose phosphate from the degradation of starch will further experience glycolysis. Totally, 22 enzymes that catalyze all the steps in the glycolysis pathway were detected, and most of them were up-regulated at 12 h imbibition. Pyruvate, the final product of glycolysis, could be further degraded through TCA cycle; most TCA cycle-related proteins were also identified as up-regulated proteins (Yang et al., 2007). Kim et al. (2009) had detected that both succinyl-CoA ligase and cytoplasmic malate dehydrogenase were stably accumulated during germination. TCA cycle might, along with glycolysis, provide the main energy at the late stage of germination. Due to lack of functional mitochondria, the aerobic respiration was detected very low during the first 48 h of imbibition. Anaerobic respiration pathway, such as fermentation, might be the main source of energy at the early stage of germination, which is supported by the identification of lactate dehydrogenase (LDH), pyruvate decarboxylase and alcohol dehydrogenase during this period (He et al., 2011a,b; Yang et al., 2007). Most of the enzymes involved in the PPP also existed in the germinating seeds, and the glycolytic enzymes were identified to be carbonylated and degraded during germination (Job et al., 2005). Blocking glycolysis and reorienting the glucose flux to the PPP could provide cells not only with reducing power in the form of NADPH to surmount oxidative stress, but also pentose phosphate for nucleotide metabolism (Arc et al., 2011).

To be mentioned, all the enzymes related to starch biosynthesis also exist in the embryo of germinating rice seeds. The accumulation of starch granules had been observed in rice embryos during germination, which suggested the solute from the endosperm can be re-synthesized to starch at the embryo (Matsukura et al., 2000; He et al., 2011a,b). But how those solutes were transferred from endosperm to embryo is not understood well. To reboot the quiescent system, *de novo* synthesis of new functional proteins is necessary. Enzymes that related to protein biosynthesis, modification, targeting, and folding, including ribosomal proteins, translation initiation or elongation factors, amino acid activation proteins, trafficking, and secretion proteins and chaperonins, were identified during germination. On the contrary, the storage proteins, seed maturation associated proteins, embryogenesis proteins and proteins related to desiccation were degraded to provide primary amino acids and reduced nitrogen for the seed germination. Although the lipids are not the major reserves, the storage lipids did accumulate during the grain filling and stored in aleurone cells of the cereal seeds (Krishnan and Dayanandan, 2003). β-oxidation and glyoxylate cycle are the two major fatty acids degradation pathways. Enzymes involved in these two pathways were detected during rice seed germination. Interestingly, fatty acid biosynthesis related enzymes such as ATP: citrate lyase and some transacylase had also been detected. Most of amino acids biosynthesis and degradation related proteins also had been identified during rice seed germination. Since most of the biosynthesis and degradation metabolisms are in concurrence, we may wonder when storage reserves should be mobilized during seed germination. Gallardo et al. (2001) inferred that the potential mobilization might exist not only in germination but also in the maturation phase based on the fact that both precursor forms and proteolyzed forms of the 12S seed storage-protein subunit were identified in dry mature seeds.

Reactive oxygen species (ROS) are produced in all living organisms. The ROS might be bifunctional, and over accumulation of ROS can result in oxidative stress. Reducing the oxidized proteins is another critical way to cope with ROS. Upon imbibition, the contents of ROS were gradually increased. ROS can be efficiently scavenged by the antioxidant enzymes like superoxide dismutases (SODs), glutathione S-transferase (GST), catalase, peroxidases, and enzymes in the ascorbate–glutathione cycle. Many of these redox regulation proteins were identified during germination (He et al., 2011a). Carbonylation, one of the important post-translational modifications (PTMs) under oxidative stress (Nystrom, 2005), can not only provoke the degradation of reserve proteins, but also be fatal by targeting those physiological important proteins (Job et al., 2005). Meanwhile, accumulation of NO upon seed imbibition can help to regulate the redox homeostasis by S-nitrosylation of the critical protein thiols and protect them from oxidation (Lounifi et al., 2013). So keeping the redox homeostasis is necessary *in vivo*.

Stress responsive proteins were accumulated during the seed maturation in order to survive from dessication. Among them, HSPs were the largest group. Proteins including universal stress proteins (USP), dehydrins, DNA J family, and late embryogenesis abundant (LEA) proteins can also be regarded as desiccation stress responsive proteins (Yang et al., 2007; He et al., 2011b). The metabolism of nucleotides was not active at early stage of germination since few enzymes required for nucleotide metabolism were detected. However, all 8 except proteins for polygalacturonase proteins involved in the cell wall biosynthesis were identified at 24 h after imbibition, which implied cell wall biosynthesis is active during the phase II (He et al., 2011b; Sano et al., 2012).

## Challenges and perspectives

In the last two decades, great progresses have been made in rice proteomics, which provide a comprehensive snapshot on the understanding of rice development, stress tolerance, organelle, secretome, protein post translational modification (PTM), transgenic plant screening and food-safety evaluation (Agrawal et al., 2011). Among them, proteomics of rice seed germination has also been widely conducted. However, proteomics of rice seed germination is still at its initial stage, and there are still many challenges in this field. Overcoming these challenges might be able to drive the study forward.

The first one is a common challenge for the proteomic study in plant. It is the establishment of routine and reliable method for sample preparation, including tissue harvesting and protein extraction. Previously, mixed tissues were always used to substitute the specific tissue because of the limitation in different sample separation techniques, such as using bran to imitate aleurone (Ferrari et al., 2009). Since different tissues function differently during germination, it is necessary to obtain pure tissues in order to deeply understand the mechanisms. In the future, some specific tiny samples such as aleurone and scutellum might be directly harvested with laser microdissection technique (Nakazono et al., 2003). As for protein extraction, it is critical to extract maximum number of proteins with high quality. Unfortunately, because of the existence of very high abundant storage proteins and basic proteins; it is almost impossible to obtain the protein sample with full coverage (Lee et al., 2008). We would like to suggest the combination of different techniques in protein extraction.

Secondly, protein's function is correlated to its cellular location and PTM, so sub-proteomics study of different organelles or modifications might be very helpful in understanding the mechanisms of seed germination. Up-to-date, very few organelle or modified proteomics studies have been conducted in rice seed germination because of the difficulties in isolation and enrichment of subcellular compartments (Agrawal et al., 2011). Only mitochondrion proteomic studies have been reported in germinating rice seed (Howell et al., 2006, 2007). Comprehensive subcellular proteomes covering more organelles (i.e., nuclear, ER, ribosomes, and protein storage vacuole) should be implemented in combination with optimized method and high-throughput technology. FFE (Free-flow electrophoresis, Zischka et al., 2006) might be a good choice for separating different organelles on the basis of their surface charge. The PTMs can affect protein localization, complex formation, stability, and activity (Ytterberg and Jensen, 2010), and play very important roles in dormancy release and metabolism resumption. More than 300 potential PTMs *in vivo* (http://www.abrf.org/index.cfm/dm.home) have been identified, such as phosphorylation (protein function reactived related), ubiquitination (storge protein degredation related), carbonylated (reactive oxygen related) and S-nitrosylation (nitrogen species related) (Arc et al., 2011). Studies on these protein modifications will undoubtedly contribute a lot in exploring the mechanism of rice seed germination in the future.

Thirdly, huge amount of data on rice proteome have been generated in the last 10 years. However, it is still a challenge to integrate these increasing data and provide a comprehensive insight into the germination process. Comparing with those database for Arabidopsis and soybean (Agrawal et al., 2011; Ohyanagi et al., 2012), few proteome databases had been constructed on rice (Komatsu, 2005; Helmy et al., 2011). However, the database has not been updated in time. In addition, various “omic” data can also provide additive information. So it is necessary to integrate proteomic data with the available genomic, transcriptomic, and metabolomic data in order to comprehensively understand the process of germination.

## Conflict of interest statement

The authors declare that the research was conducted in the absence of any commercial or financial relationships that could be construed as a potential conflict of interest.
